# Distribution of millipedes along an altitudinal gradient in the south of Lake Teletskoye, Altai Mts, Russia (Diplopoda)

**DOI:** 10.3897/zookeys.510.8855

**Published:** 2015-06-30

**Authors:** Julia S. Nefedieva, Pavel S. Nefediev, Miroslava B. Sakhnevich, Yuri V. Dyachkov

**Affiliations:** 1 Barnaul Branch of OJSC “GIPRODORNII”, Papanintsev street 105, Barnaul, 656000, Russia; 2 Department of Ecology, Biochemistry and Biotechnology, Altai State University, Lenina avenue 61, Barnaul,656049, Russia; 3 Altai State Nature Biosphere Reserve, Naberezhnyi lane 1, Gorno-Altaisk, 649000, Russia

**Keywords:** Millipedes, Diplopoda, altitudinal gradient, Lake Teletskoye, Altai, Siberia

## Abstract

The distribution of millipedes along an altitudinal gradient in the south of Lake Teletskoye, Altai, Russia based on new samples from the Kyga Profile sites, as well as on partly published and freshly revised material (Mikhaljova et al. 2007, 2008, 2014, Nefedieva and Nefediev 2008, Nefediev and Nefedieva 2013, Nefedieva et al. 2014), is established. The millipede diversity is estimated to be at least 15 species and subspecies from 10 genera, 6 families and three orders. The bulk of species diversity is confined both to low- and mid-mountain chern taiga forests and high-mountain shrub tundras, whereas the highest numbers, reaching up to 130 ind./m², is shown in subalpine *Pinus sibirica* sparse growths. Based on clustering studied localities on species diversity similarity two groups of sites are defined: low-mountain sites and subalpine sparse growths of *Pinus sibirica* ones.

## Introduction

This paper continues ecological researches on the Altai millipede fauna in the south of Lake Teletskoye, Russian Altai (Nefedieva and Nefediev 2008, Nefediev and Nefedieva 2013). Some faunistic records of *Sibiriulus altaicus* Gulička, 1972 and specimens of the family Diplomaragnidae from the study localities have been made earlier (Mikhaljova et al. 2007, 2008, 2014). A brief historical account of Altai millipede fauna research can be obtained from the publication of Mikhaljova et al. (2008) and Nefediev and Nefedieva (2013).

Since 1998 the Altai State Nature Biosphere Reserve and a buffer zone around Lake Teletskoye are inscribed as one of three separate areas of UNESCO World Natural Heritage Site under the name of «Golden Mountains of Altai». Lake Teletskoye, being the deepest and the largest body of freshwater in southwest Siberia, exerts a great warming influence on local climate, in its southern part especially. Situated at a height of 435 m above sea level, the lake lies between the mountain ridges of Altyntu and Korbu, and the Chulyshman river highlands in the south.

The aim of our present paper is to explore the distribution of millipedes along an altitudinal gradient in the south of the Teletskoye Lake in the Kyga Biogeocenosis Profile.

## Material and methods

Material was collected by the first and the second authors of this article in August 2005 using hand sampling from the litter and standard technique of soil sampling (Ghilarov 1987): 8 soil samples per each studied numbered site, sample area ¼ m^2^, depth 10 cm. The total amount of studied millipedes is 968 specimens.

The Kyga Biogeocenosis Profile was laid in 1959–1961 in the territory of watershed of the Kyga and Bayas rivers in the south of the Teletskoye Lake near the cordon of Chiri, Ulagan District, Republic of Altai, Siberia, Russia. The profile encompasses 22 numbered sites at different altitudes, and comprising relic *Pinus sibirica* forests from the Tertiary period. Its length is about 12 km, and its altitude ranges from 443 to 2267 m a.s.l. It is begun at the mouth of the Kyga river and ends on the top of the Malaya Koliushta mountain. The vertical vegetation zonation here is characterized by the presence of forest and high-mountain belts. There are widespread dark coniferous forests with *Betula pendula* and *Populus tremula*, also called as chern taiga, and sparse growths of *Pinus sibirica* in the former belt, whereas alpine meadows do not occur almost at all in the latter. Above the timberline at a height of 2100 m above sea level, all hilltops are occupied by shrub, moss-lichen and rocky tundras.

All sites we collected are listed and described below according to the following standard: site number (bold): GPS (WGS84) position, altitude, habitat, sampling date, sampling methods.

**1**: 51°20'47,3"N, 87°51'14,2"E, 443 m a.s.l., *Pinus sylvestris* and *Betula pendula* forest with *Larix sibirica*, *Abies sibirica* and *Pinus sibirica*, 12.08.2005, soil sampling.

**2**: 51°20'29,3"N, 87°51'40,0"E, 494 m a.s.l., *Abies sibirica* and *Pinus sibirica* forest with ferns, 12.08.2005, soil sampling.

**4**: 51°19'53,3"N, 87°51'78,0"E, 675 m a.s.l., *Abies sibirica* forest with *Pinus sibirica* and *Betula pendula*, 18.08.2005, soil sampling.

**5**: 51°19'28,5"N, 87°52'4,8"E, 853 m a.s.l., *Abies sibirica* forest with *Pinus sibirica*, *Populus tremula* and *Betula pendula*, 18.08.2005, soil sampling and hand sampling.

**6**: 51°19'31,6"N, 87°52'16,1"E, 942 m a.s.l., *Populus tremula* forest with *Abies sibirica* and *Pinus sibirica*, 18.08.2005, soil sampling and hand sampling.

**7**: 51°19'31,2"N, 87°52'21,1"E, 960 m a.s.l., *Abies sibirica*, *Pinus sibirica* and *Populus tremula* forest, 18.08.2005, soil sampling and hand sampling.

**8**: 51°19'30,4"N, 87°52'50,0"E, 1100 m a.s.l., *Pinus sibirica* forest with *Abies sibirica*, 17.08.2005, soil sampling.

**8a**: 51°19'23,6"N, 87°53'2,1"E, 1191 m a.s.l., *Pinus sibirica* forest with *Abies sibirica*, 17.08.2005, soil sampling and hand sampling.

**9**: 51°19'07,5"N, 87°53'15,0"E, 1414 m a.s.l., *Pinus sibirica* forest with *Abies sibirica*, 17.08.2005, soil sampling.

**10**: 51°18'58,5"N, 87°53'33,3"E, 1468 m a.s.l., *Pinus sibirica* forest with *Abies sibirica*, 17.08.2005, soil sampling.

**10a**: 51°18'43,7"N, 87°54'23,7"E, 1699 m a.s.l., sparse growths of *Pinus sibirica* with *Betula pendula* and *Abies sibirica*, 16.08.2005, soil sampling.

**11**: 51°18'41,3"N, 87°55'34,7"E, 1736 m a.s.l., old fire-site, *Betula rotundifolia* and *Salix glauca* bushes with *Pinus sibirica* and *Abies sibirica* sparse growths, 16.08.2005, soil sampling.

**12**: 51°18'27,8"N, 87°54'57,4"E, 1847 m a.s.l., old fire-site, *Betula rotundifolia* and *Salix glauca* bushes with *Pinus sibirica* and *Abies sibirica* sparse growths, 16.08.2005, soil sampling and hand sampling.

**13**: 51°18'09,4"N, 87°55'43"E, 1861 m a.s.l., subalpine *Pinus sibirica* forest, 14.08.2005, hand sampling.

**13a**: 51°18'24,1"N, 87°55'06,9"E, 1877 m a.s.l., subalpine sparse growths of *Pinus sibirica*, 16.08.2005, soil sampling and hand sampling.

**14**: 51°18'23,0"N, 87°55'22,1"E, 1903 m a.s.l., subalpine sparse growths of *Pinus sibirica*, 14.08.2005, soil sampling.

**15**: 51°18'24,5"N, 87°55'31,0"E, 1962 m a.s.l., subalpine sparse growths of *Pinus sibirica* with *Abies sibirica*, 14.08.2005, soil sampling and hand sampling under stones.

**16**: 51°18'33,6"N, 87°55'32,9"E, 2028 m a.s.l., subgoltsy sparse growths of *Pinus sibirica* with *Betula rotundifolia* and *Salix glauca* bushes, 15.08.2005, soil sampling and hand sampling.

**18**: 51°18'30,0"N, 87°56'10,7"E, 2194 m a.s.l., *Betula rotundifolia* and *Salix glauca* mountain tundra with *Dryas* and lichens, 15.08.2005, soil sampling and hand sampling under stones.

**19**: 51°18'30,5"N, 87°56'21,7"E, 2267 m a.s.l., summit of Malaya Koliushta Mt., *Betula rotundifolia* and *Salix glauca* rocky mountain tundra with *Dryas*, *Festuca* and lichens, 15.08.2005, soil sampling and hand sampling under stones.

Also we collected some material from two additional sampling sites (not included to official list of profile’s sites):

**A**: 51°20'16,8"N, 87°51'47,6"E, about 500 m a.s.l., *Duschekia fruticosa* forest on the bank of the river Bayas, 12.08.2005, hand sampling.

**B**: 51°18'05,4"N, 87°55'48,3"E, about 1900 m a.s.l., *Pinus sibirica* sparse growths, 14.08.2005, hand sampling.

The cluster analysis was performed using Statistica 10 (StatSoft 2011).

The material treated herein has been deposited mainly in the collection of the Altai State University, Barnaul, Russia (ASU), and partly shared also with the collection of the Institute of Biology and Soil Science, Far Eastern Branch, Russian Academy of Sciences, Vladivostok, Russia (IBSS), as indicated in the text. The species names include the literature references concerning Asian Russia only.

## Taxonomic part

### Order Julida Brandt, 1833

#### Family Julidae Leach, 1814

##### Genus *Julus* Linnaeus, 1758

###### Julus ghilarovi ghilarovi

Taxon classificationAnimaliaJulidaJulidae

Gulička, 1963

Julus ghilarovi Gulička, 1963: 521, 520: figs.
Julus ghilarovi – Mikhaljova 2002: 206; Nefediev 2002b: 35; Mikhaljova and Nefediev 2003: 84.
Julus ghilarovi ghilarovi – Lokšina and Golovatch 1979: 386; Mikhaljova 1993: 11: figs; 2004: 59–61, 60: figs, 61: map; 2013a: 8; Mikhaljova and Golovatch 2001: 104; Nefediev 2005a: 41; 2005b: 8; Nefediev and Nefedieva 2006: 98; 2007a: 139; 2007b: 161; 2008a: 117; 2008b: 62; 2013: 87; Nefedieva and Nefediev 2008: 123; Babenko et al. 2009: 183; Nefediev et al. 2014: 63; Nefedieva et al. 2014: 65.

####### Material examined.

1 male, 7 females, 10 juv. (ASU), site 1; 1 male, 4 females, 3 juv. (ASU), site A; 1 female (IBSS), site 4; 5 males, 12 females, 4 juv. (ASU), site 5; 4 males, 6 females, 5 juv. (ASU), site 6; 1 female, 3 juv. (ASU), site 7; 3 juv. (ASU), site 8; 1 male, 1 female (IBSS), 3 males, 8 females, 11 juv. (ASU), site 19.

####### Distribution.

This species appears to be widespread in the south of Siberia, Russia: Novosibirsk Area, Kemerovo Area, Altai Province, Republic of Altai, Republic of Khakassia, southern part of Krasnoyarsk Province. It is very likely it also occurs in the adjacent part of the Republic of Tyva.

####### Remarks.

High ecological plasticity of this species allows it to inhabit different habitats like small-leaved, mixed and dark coniferous forests, herbaceous and alpine meadows, and montane moss-stony tundras. In the Kyga Biogeocenosis Profile the animal prefers forest litter in low- and mid-mountain chern taiga forests up to about 1200 m a.s.l., and also recorded in rocky mountain tundra on the summit of Malaya Koliushta Mt. at 2267 m a.s.l. It is very likely that the species is displaced from subalpine sparse growths of *Pinus sibirica* by the congener of *Julus insolitus*. The numbers range from 0.5 to 18 ind./m^2^.

###### Julus insolitus

Taxon classificationAnimaliaJulidaJulidae

Mikhaljova, 2009

non Julus ghilarovi brachydactylus – Nefedieva and Nefediev 2008: 123.
Julus insolitus Mikhaljova, 2009b: 66–67, 64: figs.
Julus insolitus – Nefediev and Nefedieva 2013: 87; Nefedieva et al. 2014: 65.

####### Material examined.

4 males, 5 females, 4 juv. (ASU), site 9; 4 males, 1 female (IBSS), 6 males, 9 females, 10 juv. (ASU), site 10; 2 males, 6 females, 17 juv. (ASU), site 10a; 7 males, 17 females, 25 juv. (ASU), site 11; 20 males, 9 females, 49 juv., 1 fragm. (ASU), site 12; 1 male, 1 female, 1 juv. (ASU), site 13; 2 males, 4 females, 7 juv. (ASU), site 13a; 3 males, 8 females, 10 juv. (ASU), site 14; 1 female, 2 juv. (ASU), site 15; 14 males, 15 females, 22 juv. (ASU), site B; 10 males, 12 females, 15 juv. (ASU), site 16; 2 females, 6 juv. (ASU), site 18.

####### Distribution.

The species appears to be spread only in the south of Siberia, Russia: Republic of Altai.

####### Remarks.

This species has been described by Mikhaljova (2009b) on two male specimens, which are known to occur in forest-tundra and rocky tundra. In the Kyga Biogeocenosis Profile the species is collected from the upper line of mid-mountain chern taiga forests through subalpine sparse growths of *Pinus sibirica* to mountain tundra with *Betula rotundifolia* and *Salix glauca*. The maximum abundance is about 98 ind./m^2 ^registered in the old fire-site of sparse growths of *Pinus sibirica* now occupied with a succession of dwarf trees of *Betula rotundifolia* and *Salix glauca*. The above female specimens are the first records in this species.

##### Genus *Pacifiiulus* Mikhaljova, 1982

###### Pacifiiulus amurensis

Taxon classificationAnimaliaJulidaJulidae

(Gerstfeldt, 1859)

Julus amurensis Gerstfeldt, 1859: 271.
Julus amurensis – Lokšina and Golovatch 1979: 387; Mikhaljova 1993: 34.
Pacifiiulus imbricatus Mikhaljova, 1982: 211, 212: figs.
Pacifiiulus imbricatus – Mikhaljova 1983: 87; 1988: 70; Mikhaljova and Petukhova 1983: 53; Ganin 1988: 7; 1989: 145; 1994: 60; 1995: 370; 1997: 10; Ryabinin et al. 1988: 31; Mikhaljova and Bakurov 1989: 40; Gromyko 1990: 66; Mikhaljova 1993: 12: map; 1997: 145; Enghoff 1994: 27; Shelley et al. 2000: 50.
Pacifiiulus amurensis – Mikhaljova 1998a: 5; 1998b: 64: figs, 65: map; 2004: 66–69, 67: figs, 68: map; 2009a: 603; 2009c: 3; 2012a: 23; 2012b: 112; Mikhaljova and Golovatch 2001: 105; Mikhaljova and Nefediev 2003: 84; Mikhaljova and Marusik 2004: 3; Nefediev 2005a: 48; 2005b: 8; Nefediev and Nefedieva 2006: 98; 2007a: 139; 2007b: 160; 2008b: 62; 2013: 87; Nefedieva et al. 2014: 65.

####### Material examined.

1 male (IBSS), site 9; 3 males (ASU), site 16; 1 male (ASU), site 18.

####### Distribution.

This species is characterized by disjunctive area. The first distribution area is in the south of Siberia (Republic of Altai, Republic of Khakassia, southern part of Krasnoyarsk Province, Republic of Tyva) and the second one spreads in the Russian Far East (Maritime Province, southern part of Khabarovsk Province, Amur Area, Jewish Autonomous Area) and North-Eastern China.

####### Remarks.

This species is characterized by euryoky, dwelling in Siberia in herbaceous meadows, small-leaved, mixed and dark coniferous forests, and subalpine meadows and montane tundras, up to 2500 m a.s.l. (Mikhaljova and Nefediev 2003). In the Kyga Biogeocenosis Profile the species is very rare (0.5–1.5 ind./m^2^), collected from the mid-mountain dark coniferous forest, and also from subgoltsy sparse growths of *Pinus sibirica* and mountain tundra with dwarf bushes of *Betula rotundifolia* and *Salix glauca*, with the maximum altitude registered is about 2194 m a.s.l.

##### Genus *Sibiriulus* Gulička, 1963

###### Sibiriulus altaicus

Taxon classificationAnimaliaJulidaJulidae

Gulička, 1972

Cylindroiulus (Sibiriulus) altaicus Gulička 1972: 43–44, 44: fig.
Sibiriulus altaicus – Lokšina and Golovatch 1979: 387; Mikhaljova 1993: 13; 2004: 75: fig, 74: map; Mikhaljova and Golovatch 2001: 106; Mikhaljova et al. 2007: 57–59, 62, 58: figs; 2014: 45–47, 46: figs; Nefediev and Nefedieva 2007b: 162; 2008a: 117; 2008b: 62; 2013: 86–87; Nefedieva and Nefediev 2008: 123–124; Nefedieva et al. 2014: 65.

####### Material re-examined

(specimens published by Mikhaljova et al. 2007, 2014). 1 male (ASU), site 1; 3 males, 2 juv. (IBSS), site 4; 1 male, 1 female, 2 juv. (IBSS), site 7; 1 male (ASU), site 8; 1 female (IBSS), 1 female (ASU), site 8A; 1 male (ASU), site 9; 1 male (IBSS), site 14.

####### Distribution.

The species is known only in the south of Lake Teletskoye, Republic of Altai, its terra typica.

####### Remarks.

In the Kyga Biogeocenosis Profile this species dwells in low- and mid-mountain chern taiga forests, and also subalpine sparse growths of *Pinus sibirica*, with the maximum altitude registered is about 1903 m a.s.l.

#### Family Nemasomatidae Bollman, 1893

##### Genus *Orinisobates* Lohmander, 1933

###### Orinisobates sibiricus

Taxon classificationAnimaliaJulidaNemasomatidae

(Gulička, 1963)

Isobates sibiricus Gulička, 1963: 522: figs.
Isobates (Orinisobates) sibiricus – Gulička 1972: 45: figs; Nefediev and Nefedieva 2008a: 117.
Orinisobates sibiricus – Lokšina and Golovatch 1979: 387; Enghoff 1985: 53, 54: figs; Mikhaljova 1993: 16; 2002: 206; 2004: 96–97, 96: figs, 94: map; Mikhaljova and Golovatch 2001: 107; Mikhaljova and Nefediev 2003: 83; Nefediev 2005a: 39; 2005b: 8; Nefediev and Nefedieva 2006: 98; 2007a: 139; 2007b: 160; 2008a: 117; 2008b: 62; 2013: 87; Nefedieva and Nefediev 2008: 123; Nefediev et al. 2014: 63; Nefedieva et al. 2014: 65.

####### Material examined.

1 male (ASU), site 1; 1 female (ASU), site 8a.

####### Distribution.

The species appears to be quite widespread in the south of Siberia, Russia: Kemerovo Area, Republic of Khakassia, Altai Province, Republic of Altai, southern part of Krasnoyarsk Province, Republic of Tyva, Chita Area. Also it has been recorded in Eastern Kazakhstan and Kyrgyzstan.

####### Remarks.

The species inhabits forest litter of small-leaved, mixed and dark coniferous forests, under bark of logs and trees, and in mosses and mushrooms. The maximum altitude registered is about 1700 m a.s.l. (Mikhaljova and Golovatch 2001). In the Kyga Biogeocenosis Profile the species is very rare collected from low- and mid-mountain chern taiga forest, with the maximum abundance registered is about 1 ind./m^2^.

#### Julidae gen. sp.

**Material examined.** 1 juv. (ASU), site 2; 2 females, 1 juv. (ASU), site 9; 1 female, 2 juv. (ASU), site 10.

**Remarks.** It is very likely these females and juveniles appear to belong to *Sibiriulus altaicus* or *Pacifiiulus amurensis*.

### Order Chordeumatida C. L. Koch, 1847

#### Family Diplomaragnidae Attems, 1907

##### Genus *Altajosoma* Gulička, 1972

###### Altajosoma bakurovi bakurovi

Taxon classificationAnimaliaChordeumatidaDiplomaragnidae

(Shear, 1990)

Diplomaragna bakurovi Shear, 1990: 22, 23: figs.
Diplomaragna bakurovi – Mikhaljova 1993: 18.
Altajosoma bakurovi – Mikhaljova 2000: 161: fig; 2004: 178–179, 178: figs, 116: map; Mikhaljova and Golovatch 2001:108; Nefediev 2002: 30; Mikhaljova and Nefediev 2003: 86; Mikhaljova et al. 2008: 51; Nefedieva and Nefediev 2008: 123–124; 2013: 87; Nefedieva et al. 2014: 65.

####### Material re-examined

(specimen published by Mikhaljova et al. 2008). 1 male (ASU), site A.

####### Distribution.

The species is known to occur in the south of Siberia, Russia: Tomsk, Novosibirsk and Kemerovo areas, Krasnoyarsk Province and Republic of Altai.

####### Remarks.

This species dwells in various forest habitats like small-leaved, mixed and dark coniferous forests, and also mesophytous meadow and mountain tundra, with the maximum altitude registered is about 2500 m a.s.l (Mikhaljova and Nefediev 2003). In the Kyga Biogeocenosis Profile a single male is collected only by hand sampling from *Duschekia fruticosa* forest on the bank of the river Bayas at about 500 m a.s.l.

###### Altajosoma deplanatum

Taxon classificationAnimaliaChordeumatidaDiplomaragnidae

(Stuxberg, 1876)

Craspedosoma deplanatum Stuxberg, 1876a: 34, figs.
Craspedosoma deplanatum – Stuxberg 1876b: 317; Lokšina and Golovatch 1979: 382; Nefediev and Nefedieva 2008a: 117.
Altajosoma pinetorum Gulička, 1972: 37: figs.
Altajosoma pinetorum – Lokšina and Golovatch 1979: 382; Shelley et al. 2000: 62; Nefediev and Nefedieva 2008a: 117.
Diplomaragna deplanata – Shear 1990: 19, 20: figs; Mikhaljova 1993: 22.
Diplomaragna pinetorum – Shear 1990: 38; Mikhaljova 1993: 25.
Altajosoma deplanatum – Mikhaljova 2000: 160; 2004: 170–171, 171: figs, 162: map; 2013a: 7; Mikhaljova and Golovatch 2001: 108; Nefediev 2002b: 35; 2002d: 30; Mikhaljova and Nefediev 2003: 86; Nefediev 2005a: 50; 2005b: 9; Nefediev and Nefedieva 2005: 177; 2006: 98; 2007a: 139; 2007b: 161; 2007c: 99; 2008b: 62; 2011: 100; 2012a: 51; 2012b: 47; 2013: 87; Nefedieva and Nefediev 2008: 123; Mikhaljova et al. 2008: 51; Nefedieva et al. 2014: 65.

####### Material examined.

2 females (ASU), site 14.

####### Material re-examined

(specimens published by Mikhaljova et al. 2008). 1 male (ASU), site 6; 1 male, 6 juv. (ASU), site 14.

####### Distribution.

The species appears to be quite widespread in the south of Siberia, Russia: Tomsk, Novosibirsk and Kemerovo areas, Republic of Altai and Republic of Khakassia, and originally described from between the city of Achinsk (Krasnoyarsk Province) and the city of Mariinsk (Kemerovo Area).

####### Remarks.

This species lives mainly in various forest habitats like small-leaved, mixed and dark coniferous forests, forest- and shrub tundra, and also mesophytous meadow. The maximum altitude registered is about 2080 m a.s.l (Mikhaljova et al. 2008). In the Kyga Biogeocenosis Profile the species quite rare found in the mid-mountain mixed forest and in the subalpine sparse growths of *Pinus sibirica*, with the maximum abundance registered is about 6 ind./m^2^.

###### Altajosoma katunicum

Taxon classificationAnimaliaChordeumatidaDiplomaragnidae

Mikhaljova, 2000

Altajosoma katunicum Mikhaljova, 2000: 161–162, 162: figs.
Altajosoma katunicum – Mikhaljova and Golovatch 2001: 108; Mikhaljova 2004: 176–177, 177: figs, 112: map; Mikhaljova et al. 2008: 52; Nefedieva et al. 2014: 65.

####### Material re-examined

(specimens published by Mikhaljova et al. 2008). 1 male (ASU), site 1.

####### Distribution.

This species in known to occur only in the Republic of Altai, Russia.

####### Remarks.

This species originally described from the Katunskii Mt. Range, Central Altai at 1600–2200 m a.s.l. (Mikhaljova 2000). In the Kyga Biogeocenosis Profile the species is very rare (1 ind./m^2^), collected only from the mixed forest in the lowest study site at 443 m a.s.l.

###### Altajosoma kemerovo

Taxon classificationAnimaliaChordeumatidaDiplomaragnidae

(Shear, 1990)

Diplomaragna kemerovo Shear, 1990: 21, 20: figs.
Diplomaragna kemerovo – Mikhaljova 1993: 25; Nefediev and Nefedieva 2008a: 117.
Altajosoma kemerovo – Mikhaljova 2000: 161; 2004: 180–181, 180: figs, 173: map; 2013a: 7; Mikhaljova and Golovatch 2001: 108; Vorobiova et al. 2002: 60; Mikhaljova and Nefediev 2003: 86; Nefediev 2005a: 53; 2005b: 9; Nefediev and Nefedieva 2006: 98; 2007a: 139; 2007b: 161; 2008b: 62; 2013: 87; Nefedieva and Nefediev 2008: 123; Mikhaljova et al. 2008: 51; Nefedieva et al. 2014: 65.

####### Material examined.

4 females (ASU), site 7; 1 juv. (ASU), site 8; 7 juv. (ASU), site 13a; 1 female, 3 juv. (ASU), site 16.

####### Material re-examined

(specimens published by Mikhaljova et al. 2008). 1 male (ASU), site A; 1 male (ASU), site 5; 1 male (ASU), site 6; 5 males (ASU), site 7; 1 male (ASU), site 8; 1 male (ASU), site 13a; 1 male (ASU), site 16.

####### Distribution.

This species appears to be spread in the south of Siberia, Russia: Kemerovo and Novosibirsk areas, Republic of Altai, Republic of Khakassia, southern part of Krasnoyarsk Province.

####### Remarks.

The species dwells in different forest habitats like small-leaved, mixed and dark coniferous forests. In the Kyga Biogeocenosis Profile it is very rare (0.5–5 ind./m^2^), mainly collected in low- and mid-mountain chern taiga at 853–1100 m a.s.l., but also found in subalpine sparse growths of *Pinus sibirica* and subgoltsy sparse growths of *Pinus sibirica* with *Betula rotundifolia* and *Salix glauca* bushes, with the maximum altitude registered is about 2028 m a.s.l.

###### Shearia teletskaya

Taxon classificationAnimaliaChordeumatidaDiplomaragnidae

Mikhaljova, 2000

Shearia teletskaya Mikhaljova, 2000: 167–168, 167: figs.
Shearia teletskaya – Mikhaljova and Golovatch 2001: 111; Mikhaljova 2004: 160–161, 160: figs, 112: map; Mikhaljova et al. 2008: 54; Nefedieva et al. 2014: 65.

####### Material examined.

1 juv. (ASU), site 16.

####### Material re-examined

(specimens published by Mikhaljova et al. 2008). 1 male, 4 juv. (ASU), site B; 1 male, 8 juv. (ASU), site 12; 1 male (IBSS), 1 male (ASU), site 16.

####### Distribution.

The species is known to occur only in the south of Lake Teletskoye, Republic of Altai, Russia.

####### Remarks.

This species inhabits dark coniferous taiga forests at 1350–1750 m a.s.l. and the subalpine belt (= goltsy) at 1750–2000 m a.s.l. (Mikhaljova 2004). In the Kyga Biogeocenosis Profile sites investigated the species is very rare (3 ind./m^2^), mainly collected by hand sampling in subalpine sparse growths of *Pinus sibirica* at 1847–2028 m a.s.l.

##### Diplomaragnidae gen. sp.

**Material examined.** 1 female (ASU), site A; 1 female, 1 juv. (ASU), site 5; 5 juv. (ASU), site 8a; 4 juv. (ASU), site 9; 1 female, 14 juv. (ASU), site 10; 5 juv. (ASU), site 10a; 1 female, 9 juv. (ASU), site 11; 1 female, 1 fragm. (ASU), site 13; 1 female, 1 juv. (ASU), site 18; 1 female, 1 juv. (ASU), site 19.

**Remarks.** The above specimens appear to belong to some species of *Altajosoma* or *Shearia teletskaya*.

#### Family Anthroleucosomatidae Verhoeff, 1899

##### Genus *Ghilarovia* Gulička, 1972

###### Ghilarovia kygae

Taxon classificationAnimaliaChordeumatidaAnthroleucosomatidae

Gulička, 1972

Ghilarovia kygae Gulička, 1972: 39, 40: figs.
Ghilarovia kygae – Lokšina and Golovatch 1979: 383; Shear 1988: 55: figs; Mikhaljova 1993: 16; 2002: 203, 202: figs; 2004: 188–190,188: figs, 189: map; 2013a: 8; Shelley et al. 2000: 68; Mikhaljova and Golovatch 2001: 107; Nefediev 2005a: 58; 2005b: 9; Nefediev and Nefedieva 2007b: 161; 2008a: 117; 2008b: 62; 2013: 87; Nefedieva and Nefediev 2008: 123; Nefedieva et al. 2014: 65.

####### Material examined.

18 males, 16 females (ASU), site 1; 9 males, 6 females (ASU), site 2; 2 males, 1 female (ASU), site A; 3 males, 9 females, 2 juv. (ASU), site 4; 24 males, 18 females, 2 juv. (ASU), site 5; 15 males, 15 females, 2 juv. (ASU), site 6; 21 males, 15 females, 1 juv., 1 fragm. (ASU), site 7; 5 males, 3 females (ASU), site 8; 6 males, 6 females, 14 juv. (ASU), site 8a; 2 females, 1 juv. (ASU), site 9; 2 males (ASU), site 10a; 3 males, 3 females (ASU), site 11; 3 males, 1 female, 8 juv. (ASU), site 12; 3 males, 1 female (ASU), site 12; 6 males, 8 females (ASU), site 13; 2 males, 5 females (ASU), site 13a; 5 males, 16 females (ASU), site 14; 1 male, 1 female, 1 fragm. (ASU), site 15; 2 males, 8 females (ASU), site B; 1 male (ASU), site 16.

####### Distribution.

The species is known to occur in the Republic of Altai, Siberia, Russia only.

####### Remarks.

This species prefers to live in mixed and dark coniferous forests, and also known from subalpine habitats like golsty, with the maximum altitude registered is about 2000 m a.s.l. (Mikhaljova and Golovatch 2001). In the Kyga Biogeocenosis Profile the species shows the highest ecological plasticity, dwelling in low- and mid-mountain chern taiga forests, and subalpine sparse growths of *Pinus sibirica* up to subgoltsy, with the maximum altitude registered is about 2028 m a.s.l.

##### Family Kirkayakidae Özdikmen, 2008

(syn. Altajellidae Mikhaljova & Golovatch, 2001)

##### Genus *Kirkayakus* Özdikmen, 2008

(syn. *Altajella* Gulička, 1972)

###### Kirkayakus pallidus

Taxon classificationAnimaliaChordeumatidaAnthroleucosomatidae

(Gulička, 1972)

Altajella pallida (syn. Gulička, 1972)
Altajella pallida Gulička, 1972: 42, 43: figs.
Altajella pallida – Lokšina and Golovatch 1979: 383; Shear 1988: 51; Mikhaljova 1993: 34; 2004: 196–199, 197: figs, 105: map; Shelley et al. 2000: 61; Mikhaljova and Golovatch 2001: 111, 112: figs; Nefediev 2005a: 58; 2005b: 9; Nefediev and Nefedieva 2007b: 161; 2008a: 117; 2008b: 62; Nefedieva and Nefediev 2008: 123.
Kirkayakus pallidus – Özdikmen 2008: 342; Nefediev and Nefedieva 2013: 87; Nefedieva et al. 2014: 65.

####### Material examined.

1 male (ASU), site 8; 1 female (ASU), site 9.

####### Distribution.

This species is an endemic in the south of Lake Teletskoye, Republic of Altai, Siberia, Russia.

####### Remarks.

The species lives in dark coniferous forests up to 1350 m a.s.l. (Mikhaljova and Golovatch 2001). In the Kyga Biogeocenosis Profile the animal prefers mid-mountain dark coniferous forests at 1100–1414 m a.s.l., when it is very rare, and the numbers range from 0.5 to 1 ind./m^2^. The above material appears to belong to topotypes, and this is the first record of the female specimen in this species.

##### Genus *Teleckophoron* Gulička, 1972

###### Teleckophoron montanum

Taxon classificationAnimaliaChordeumatidaAltajellidae

Gulička, 1972

Teleckophoron montanum Gulička, 1972: 41: figs.
Teleckophoron montanum – Lokšina and Golovatch 1979: 383; Mikhaljova 1993: 35; 2004: 193–196, 195: figs, 107: map; Shelley et al. 2000: 79; Mikhaljova and Golovatch 2001: 113, 114: figs; Nefediev 2005a: 59; 2005b: 9; Nefediev and Nefedieva 2006: 98; 2007b: 161; 2008a: 117; 2008b: 62; 2013: 87; Nefedieva and Nefediev 2008: 123; Nefedieva et al. 2014: 65.

####### Material examined.

3 males, 1 female, 4 juv. (ASU), site 8; 1 female, 3 juv. (ASU), site 8a.

####### Distribution.

The area of this species appears to encompass the Republic of Altai and the southern part of the Krasnoyarsk Province, both Siberia, Russia.

####### Remarks.

This species inhabits dark coniferous forests and montane tundras. The maximum altitude registered is about 1000 m a.s.l. (Gulička 1972). In the Kyga Biogeocenosis Profile the species prefers mid-mountain dark coniferous forests up to 1191 m a.s.l., where the numbers range from 3 to 8 ind./m^2^.

### Order Polydesmida Leach, 1815

#### Family Polydesmidae Leach, 1815

##### Genus *Schizoturanius* Verhoeff, 1931

###### Schizoturanius clavatipes

Taxon classificationAnimaliaPolydesmidaPolydesmidae

(Stuxberg, 1876)

Polydesmus clavatipes Stuxberg, 1876a: 34, figs.
Polydesmus clavatipes – Stuxberg 1876b: 316; Nefediev and Nefedieva 2008a: 117.
Schizoturanius clavatipes – Lohmander 1933: 27; Hoffman 1975: 81, 82: figs; Lokšina and Golovatch 1979: 384; Mikhaljova 1993: 31, 32: figs; 2002: 206; 2004: 238–240, 239: figs, 228: map; 2013a: 9; 2013b: 221; Nefediev 2001: 84; 2002c: 139; 2002d: 30; Mikhaljova and Golovatch 2001: 116; Vorobiova et al. 2002: 60; Mikhaljova and Nefediev 2003: 81; Nefediev 2005a: 61; 2005b: 9; Nefediev and Nefedieva 2005: 178; 2006: 98; 2007a: 139; 2007b: 161; 2007c: 102; 2008b: 62; 2011: 100; 2012a: 51; 2012b: 47; 2013: 87; Nefedieva and Nefediev 2008: 123; Nefediev et al. 2014: 63; Nefedieva et al. 2014: 65.

####### Material examined.

4 males, 2 females, 4 juv. (ASU), site 1; 2 males, 2 females, 3 juv. (ASU), site 2; 2 males, 1 female, 1 juv. (ASU), site 4; 4 males, 2 females, 1 juv. (ASU), site 5; 2 males, 1 females (ASU), site 6; 6 males, 2 females, 11 juv. (ASU), site 7; 1 male, 1 female (ASU), site 8a; 1 female (ASU), site 9; 7 juv. (ASU), site 14; 3 juv. (ASU), site B.

####### Distribution.

This species appears to be quite widespread in the south of Siberia, Russia: Tomsk, Novosibirsk and Kemerovo areas, Altai Province, Republic of Altai, Republic of Khakassia, southern part of Krasnoyarsk Province.

####### Remarks.

Being highly euryoecic, the species populates various forest habitats (small-leaved, mixed and dark coniferous forests), and also meadows and glades. In the Kyga Biogeocenosis Profile the species prefers low- and mid-mountain chern taiga forests, where its numbers range from 0.5 to 10 ind./m^2^, but also collected from subalpine sparse growths of *Pinus sibirica* at about 1903 m a.s.l.

###### Schizoturanius tabescens

Taxon classificationAnimaliaPolydesmidaPolydesmidae

(Stuxberg, 1876)

Polydesmus tabescens Stuxberg, 1876a: 35, figs.
Polydesmus tabescens – Stuxberg 1876b: 316; Lokšina and Golovatch 1979: 385.
Turanodesmus salairicus Gulička, 1963: 523, 522: figs; Nefediev and Nefedieva 2008a: 117.
Schizoturanius salairicus – Lokšina and Golovatch 1979: 384; Mikhaljova 1993: 31; Nefediev 2001: 84, 2002a: 40; 2002b: 35; 2002c: 139; 2002d: 30; Mikhaljova and Golovatch 2001: 116; Mikhaljova and Nefediev 2003: 83.
Schizoturanius tabescens – Mikhaljova 1993: 31, 32: figs; 2004: 240–242, 241: figs, 242: map; 2013b: 221; Vorobiova 1999: 33; Mikhaljova and Golovatch 2001: 116; Vorobiova et al. 2002: 60; Rybalov 2002; Mikhaljova and Marusik 2004: 8, 7: figs; Nefediev 2005a: 64; 2005b: 9; Nefediev and Nefedieva 2005: 178; 2006: 98; 2007a: 139; 2007b: 161; 2007c: 102; 2008a: 117; 2008b: 62; 2011: 100; 2012a: 51; 2012b: 47; 2013: 87; Nefedieva and Nefediev 2008: 123; Babenko et al. 2009: 183; Nefedieva et al. 2014: 65.

####### Material examined.

3 juv. (ASU), site 2; 2 females, 2 juv., 1 fragm. (ASU), site 4; 4 juv. (ASU), site 5; 17 juv. (ASU), site 7; 2 females, 1 juv. (ASU), site 8a; 1 female (ASU), site 9; 2 females (ASU), site B.

####### Distribution.

Being rather widespread, the species is known to occur in the south of Siberia, Russia: Tomsk, Novosibirsk and Kemerovo areas, Altai Province, Republic of Altai, Republic of Khakassia, southern part of Krasnoyarsk Province.

####### Remarks.

This species lives in different forests like small-leaved, mixed and dark coniferous ones, and also populates meadows. In the Kyga Biogeocenosis Profile the animal prefers low- and mid-mountain chern taiga forests, where its numbers range from 0.5 to 8 ind./m^2^, but also collected from subalpine sparse growths of *Pinus sibirica* at about 1900 m a.s.l.

## Results

The millipede diversity in the south of Lake Teletskoye is estimated to be at least 15 species and subspecies from 10 genera, 6 families and three orders: *Julus ghilarovi ghilarovi* Gulička, 1963, *Julus insolitus* Mikhaljova, 2009, *Orinisobates sibiricus* (Gulička, 1963), *Pacifiiulus amurensis* (Gertsfeldt, 1859), *Sibiriulus altaicus* (Gulička, 1972), *Ghilarovia kygae* Gulička, 1972, *Kirkayakus pallidus* (Gulička, 1972) (synonym of *Altajella pallida* Gulička, 1972), *Teleckophoron montanum* Gulička, 1972, *Altajosoma bakurovi bakurovi* (Shear, 1990), *Altajosoma deplanatum* (Stuxberg, 1876), *Altajosoma katunicum* Mikhaljova, 2000, *Altajosoma kemerovo* (Shear, 1990), *Shearia teletskaya* Mikhaljova, 2000, *Schizoturanius clavatipes* (Stuxberg, 1876) and *Schizoturanius tabescens* (Stuxberg, 1876).

The bulk of species diversity is confined both to low- and mid-mountain chern taiga forests and high-mountain shrub tundras with *Betula rotundifolia* and *Salix glauca*, achieving from 5 to 9 species, whereas subalpine sparse growths of *Pinus sibirica* are characterized by the lowest millipede diversity, with 3 the most widespread species (Table 1). One of them, *Ghilarovia kygae*, has the maximum of ecological plasticity. This species is recorded in almost all study sites, dwelling in low- and mid-mountain chern taiga forests, and subalpine sparse growths of *Pinus sibirica* up to subgoltsy, with the maximum altitude registered is about 2028 m a.s.l.

**Table 1.** Millipede abundance (ind./m^2^) and hand sampling (+) in the Kyga Biogeocenosis Profile sites investigated.

Species	Sites																					
	1	2	A	4	5	6	7	8	8a	9	10	10a	11	12	13	13a	B	14	15	16	18	19
**Julida**																						
*Julus ghilarovi ghilarovi*	18		+	0.5	3	5	2	3														6
*Julus insolitus*										6.5	15	50	98	20	+	13	+	14	3	15.5	4	
*Sibiriulus altaicus*	1			2.5			2	1	2	0.5								1				
*Orinisobates sibiricus*	1								1													
*Pacifiiulus amurensis*										0.5										1.5	0.5	
Julidae gen. sp.		1								1.5	1.5											
**Chordeumatida**																						
*Altajosoma bakurovi bakurovi*			+																			
*Altajosoma deplanatum*						+												6				
*Altajosoma katunicum*	1																					
*Altajosoma kemerovo*			+		1.5	0.5	3	2								5				2.5		
*Shearia teletskaya*														3			+			+		
Diplomaragnidae gen. sp.			+						4	2	7.5	10	20		+						1	2
*Ghilarovia kygae*	34	15	+	7	16.5	14.5	14.5	8	22	1.5		4	12	2	+	4	+	14	2	0.5		
*Kirkayakus pallidus*								1		0.5												
*Teleckophoron montanum*								8	3													
**Polydesmida**																						
*Schizoturanius clavatipes*	10	7		2	1.5	1	8		2	0.5							+	4.5				
*Schizoturanius tabescens*		3		2.5	2		8		2	0.5							+					
**Total abundance**	**65**	**26**	-	**14.5**	**24.5**	**21**	**37.5**	**23**	**36**	**14**	**24**	**64**	**130**	**25**	-	**22**	-	**39.5**	**5**	**20**	**5.5**	**8**
**Numbers of species**	6	4	4	5	5	5	6	6	7	9	3	3	3	3	3	3	5	5	2	5	3	2

The numbers of diplopods range from 14.5 to 65 ind./m² in subzones of low- and mid-mountain chern taiga forests, and from 5.5 to 8 ind./m² in high-mountain shrub tundras (Table 1). Despite of the lowest species diversity in subalpine sparse growths of *Pinus sibirica*, millipedes show the maximum numbers, reaching up to 130 ind./m² in the old fire-site (site 11), evidently caused by the abundance of plant debris of dwarf vegetation of *Betula rotundifolia* and *Salix glauca* that appear to be more suitable for feeding of millipedes than pine litter.

**Figure 1.**
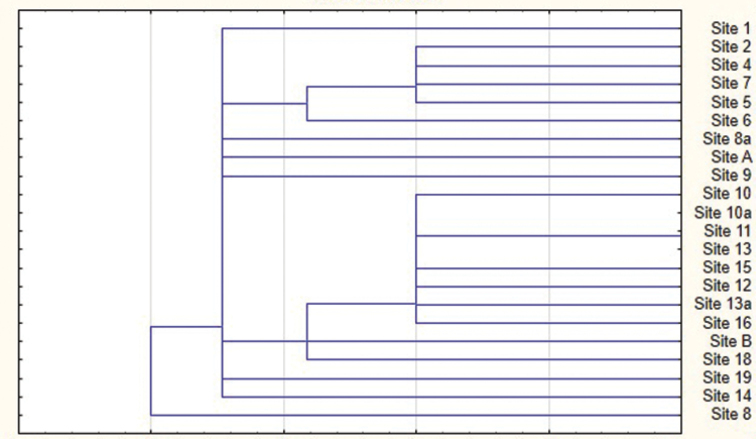
Neighbour-joining tree of similarity / dissimilarity of studied sites on species diversity.

Clustering of investigated sites in the Kyga Biogeocenosis Profile on species diversity allows to grouping at least two obvious sets of sites (Figure 1). The first group unites low-mountain chern taiga forests (sites from 2 to 7), with altitudes range from 494 to 960 m a.s.l. The second group includes subalpine sparse growths of *Pinus sibirica* localities (sites from 10a to 15), and also the highest locality of chern taiga forest (site 10) and a transition locality from subalpine sparse growths to shrub mountain tundra (site 16), with altitudes range from 1468 to 2028 m a.s.l. Some localities of mid-mountain chern taiga forests and mountain tundras are less similar both to each other and to other groups.

The very interesting situation is observed with two congeners of *Julus* as regards to altitude distribution, showing competitive relationships to each other. Thus, *Julus ghilarovi ghilarovi* is mainly recorded in low-mountain and in the beginning of mid-mountain chern taiga forests, while it almost disappears in subalpine sparse growths of *Pinus sibirica*, but it emerges again in rocky mountain tundra on the top of the investigated biogeocenosis profile. At the same time, *Julus insolitus* populates mainly subalpine sparse growths of *Pinus sibirica* and lifts up to shrub mountain tundra at 2194 m a.s.l. Taking into account that *Julus insolitus* is very abundant here, we assume it wins the competition and displaces *Julus ghilarovi ghilarovi* from subalpine *Pinus sibirica* sparse growths. The record of female specimens of *Julus insolitus* is the first one in this species.

Both members of Kirkayakidae, *Kirkayakus pallidus* and *Teleckophoron montanum*, are reported in chern mid-mountain taiga at the highest altitudes for the first time. Also this is the first record of the female specimen in the former species.

## Supplementary Material

XML Treatment for Julus ghilarovi ghilarovi

XML Treatment for Julus insolitus

XML Treatment for Pacifiiulus amurensis

XML Treatment for Sibiriulus altaicus

XML Treatment for Orinisobates sibiricus

XML Treatment for Altajosoma bakurovi bakurovi

XML Treatment for Altajosoma deplanatum

XML Treatment for Altajosoma katunicum

XML Treatment for Altajosoma kemerovo

XML Treatment for Shearia teletskaya

XML Treatment for Ghilarovia kygae

XML Treatment for Kirkayakus pallidus

XML Treatment for Teleckophoron montanum

XML Treatment for Schizoturanius clavatipes

XML Treatment for Schizoturanius tabescens
